# Determinants of early working impairments in multiple sclerosis

**DOI:** 10.3389/fneur.2022.1062847

**Published:** 2022-12-09

**Authors:** Marcello Moccia, Luca Fontana, Raffaele Palladino, Fabrizia Falco, Ferdinando Finiello, Mauro Fedele, Roberta Lanzillo, Liberata Reppuccia, Maria Triassi, Vincenzo Brescia Morra, Ivo Iavicoli

**Affiliations:** ^1^Department of Molecular Medicine and Medical Biotechnology, Federico II University of Naples, Naples, Italy; ^2^Multiple Sclerosis Clinical Unit, Federico II University Hospital, Naples, Italy; ^3^Occupational Health Unit, Department of Public Health, Federico II University of Naples, Naples, Italy; ^4^Department of Public Health, Federico II University of Naples, Naples, Italy; ^5^Department of Primary Care and Public Health, Imperial College, London, United Kingdom; ^6^Department of Neuroscience, Reproductive Science and Odontostomatology, Federico II University of Naples, Naples, Italy

**Keywords:** multiple sclerosis, working, job, disability, fatigue, mood, cognitive

## Abstract

**Introduction:**

Unemployment can directly affect social status and identity. Assessing and adjusting determinants of early working impairments in a chronic disease can thus reduce its long-term burden. Hereby, we aim to evaluate differences in occupational history and early working impairments between people with multiple sclerosis (MS) and healthy workers.

**Methods:**

This is a cross-sectional study comparing 71 workers with MS [age 41.7 ± 9.4 years; females 59.1%; EDSS 2.0 (1.0–6.0)] and 71 controls (age 42.6 ± 11.9 years; females 33.8%). All participants filled in Work Ability Index (WAI), Work Productivity and Activity Impairment (WPAI), European Questionnaire for Quality of Life (EuroQoL), Beck Depression Inventory II (BDI-II), and Pittsburgh Sleep Quality Index (PSQI). In MS, we further collected expanded disability status scale (EDSS), MS Questionnaire for Job difficulties (MSQ-Job), Fatigue severity scale (FSS), and the Brief International Cognitive Assessment for MS (BICAMS).

**Results:**

Workers with MS were more working disabled (*p* < 0.01), less exposed to workplace risks (*p* < 0.01), and more limited in fitness to work (*p* = 0.01), compared with controls. On linear regression models adjusted by age, sex, education, and type of contract, people with MS had worse WAI (Coeff=−5.47; 95% CI = −7.41, −3.53; *p* < 0.01), EuroQoL (Coeff = −4.24; 95% CI = −17.85, −6.50; *p* < 0.01), BDI-II (Coeff = 3.99; 95% CI = 2.37, 7.01; *p* < 0.01), and PSQI (Coeff = 4.74; 95% CI = 3.13, 7.61; *p* < 0.01), compared with controls, but no differences in WPAI (*p* = 0.60). EuroQoL, BDI-II, and PSQI were equally associated with both WAI and WPAI in MS and controls (all *p*< 0.01). In MS, worse MSQJob was associated with higher EDSS (Coeff = 5.22; 95% CI = 2.24, 7.95; *p* < 0.01), progressive disease (Coeff = 14.62; 95% CI = 5.56, 23.69; *p* < 0.01), EuroQoL (Coeff = 4.63; 95% CI = 2.92, 6.35; *p* < 0.01), FSS (Coeff = 0.55; 95% CI = 0.38, 0.72; *p* < 0.01), and cognitive impairment (Coeff = 4.42; 95% CI = 0.67, 8.22; *p* = 0.02).

**Discussion:**

Early factors associated with working difficulties in MS include disability, fatigue, depression, and cognitive dysfunction. Early identification of clinical features potentially causing working difficulties should be considered to enhance job retention, along with targeted prevention and protection measures.

## Introduction

Multiple sclerosis (MS) is a chronic and potentially highly-disabling disease of the central nervous system, which can lead to physical and cognitive impairment, including walking difficulties, fatigue, poor balance, bladder and bowel dysfunction, reduced visual acuity, mood changes, and impaired cognition, (1). Symptoms can present in the form of relapses, followed by a recovery period [relapsing-remitting MS (RRMS)], or as gradual progression of disability, either preceded by relapses [secondary progressive MS (SPMS)] or not [primary progressive MS (PPMS)] (2). The MS natural history has changed thanks to the use of disease modifying treatments (DMTs), which primarily target relapses, but also affect disability outcomes in the long term (3).

MS holds significant psychological, physical, financial and social burden on patients, their caregivers, and healthcare services (4–6). The heavy financial burden of MS is related to young age at onset (usually around 30 years of age), variety of chronic symptoms, and subsequently high unemployment rates (7). Indeed, about 50% of workers with MS suffer from reduced working abilities from disease onset (e.g., scaling down from full-time to part-time work), and will eventually lose (or quit) their job (8–11). Exclusion from the workplace is responsible for worsening social status and finances, thus affecting health outcomes in MS (12). The main factors potentially associated with unemployment are progressive disease course, motor disability, fatigue, mood changes, and cognitive impairments (9, 11, 13–17). Nevertheless, data on work ability and occupational difficulties related to MS are rather limited. Also, most studies compared demographic and clinical variables between employed and unemployed people with MS, with potential bias coming from largely different populations (e.g., disease duration, disability) (12). As such, in the present study, we specifically focused on workers with MS to evaluate differences in occupational history and early working impairments (e.g., ability, productivity and activity), when compared with healthy workers as controls, and to define clinical correlates of MS-related perceived working difficulties. Identifying determinants of early working impairments in employed people with MS can direct work retention strategies, ultimately reducing the long-term burden of this chronic disease.

## Methods

### Study design

This is a cross-sectional study comparing workers with MS and healthy workers as controls. The study was conducted at the MS Unit and at the Occupational Health Unit, of the Federico II University Hospital, Naples, Italy. The study was approved by Ethics Committee, at Federico II University Hospital, Naples, Italy (355/19), and all recruited subjects signed informed consent authorizing the use of anonymized data, in line with data protection regulation (GDPR EU2016/679). The study was performed in accordance with good clinical practice and Declaration of Helsinki.

### Study setting and participants

We included people with MS, consecutively recruited from Feb 2021 (until the reaching of the recruitment target, as from power calculation), according to the following inclusion criteria: (1) diagnosis of MS (18); (2) employment age (18–65 years); (3) employment in the previous 6 months; and exclusion criteria: (1) any concomitant condition, disease or treatment potentially affecting employment; (2) relapses in the previous 3 months.

We also recruited consecutively a group of healthy controls within the same age range (with a case:control matching ratio of 1:1), from Feb 2021 (until the reaching of the recruitment target, as from power calculation), while attending the same hospital within the same period for their scheduled visit at the Occupational Health Unit, in accordance with Italian legislation (Legislative Decree n. 81/2008) on the protection of health and safety in the workplace. The Occupational Health Unit regularly sees healthy workers from a number of public and private institutions, thus providing heterogeneous case mix.

Data collection was conducted by MS specialists with the support of Occupational Health specialists and neuropsychologists, as appropriate.

### Main variables of interest

To measure work ability and work productivity and activity impairment, people with MS and controls were required to fill in the following questionnaires:

- Work Ability Index questionnaire (WAI) (19), with higher scores indicating better work ability;
- Work Productivity and Activity Impairment Questionnaire: General Health questionnaire (WPAI) (20), with higher scores indicating better work productivity and activity.

To measure MS-related perceived difficulties in work-related tasks, people with MS were required to fill in the following questionnaire:

- MS Questionnaire for Job difficulties (MSQ-Job) (7), with higher percent scores indicating worse perception of difficulties in work-related tasks;

### Endpoints in people with MS and controls

We collected demographics (age, sex), education (highest educational attainment), and occupational history, using a structured questionnaire, as from the clinical practice at the Occupational Health Unit, in people with MS and controls. In detail, occupational history included the following variables: formal acknowledgment of working disability status, percent of disability status (with higher scores indicating higher disability), type of contract (e.g., permanent, temporary, self-employed), occupational risk factors (e.g., physical, ergonomic, biological, chemical, etc.), and formal limitations in the fitness to work (depending on both working disability status and exposure to specific occupational risk factors). We also classified working activity using the Italian Institute of Statistics classification (ISTAT – Nomenclatura e classificazione delle Unità Professionali), which includes: (1) law makers, businessmen, and managers; (2) intellectual, scientific and high-specialization work; (3) technical work; (4) office executive work; (5) qualified work in commercial activities and services; (6) craftsmen, skilled workers, and farmers; (7) System operators, workers of fixed and mobile machinery, and vehicle drivers; (8) Unqualified work; (9) armed forces.

Also, people with MS and controls were required to fill in the following questionnaires:

- European Questionnaire for Quality of Life – 5 Domains (EuroQoL) (21), for evaluating impairments in daily activities and percent rating of quality of life (we collected only the latter in controls, who would have had no significant impairments);
- Beck Depression Inventory II (BDI-II) (22), with higher scores indicating worse depression;
- Pittsburgh Sleep Quality Index (PSQI) (23), with higher scores indicating worse sleep quality.

MS specialists, MS nurses, and Occupational Health specialists were available to people with MS and controls while filling in the questionnaires, as needed.

### Endpoints in people with MS

In people with MS, we collected disease duration (time from reported disease onset to baseline assessment), expanded disability status scale (EDSS), clinical subtype (RRMS, SPMS and PPMS; for statistical purposes SPMS and PPMS were grouped), and DMTs (grouped into first and second line DMTs). EDSS was categorized into <3.5 and >4.0 to identify people with MS without and with walking impairment. Moreover, workers with MS were also administered the following questionnaire and neuropsychological tests:

- Fatigue severity scale (FSS) (24), with higher scores indicating worse fatigue;
- Brief International Cognitive Assessment for MS(BICAMS) neuropsychological battery, which includes the following tests: the Symbol Digit Modalities Test (SDMT), evaluating attention and information processing speed; the California Verbal Learning Test-II (CVLT-II), evaluating memory and verbal learning; and the Brief Visuospatial Memory Test-Revised (BVMTR), evaluating visuo-spatial learning (25). Results were corrected for age, sex, and education, according to the Italian normative values. We then calculated the corresponding cerebral functional system (FS) score (0 corresponds to normal BICAMS tests, ≥1 corresponds to at least one impaired BICAMS test), as from previous studies (26).

### Study size and power analysis

Considering a normal distribution of variables to be analyzed in regression model (including one dependent variable and four covariates), given a 10% minimum detectable effect size, a two-sided tail and a 5% α error, a sample of 142 individuals (71 cases and 71 controls) would be able to achieve 98% power.

### Statistical methods

Results are presented as mean (standard deviation), median (range), or number (percent), as appropriate. Differences in demographics and occupational history in people with MS and controls were evaluated using *t*-test, chi-square test and Fisher's exact test, as appropriate.

Differences between MS cases and controls were evaluated using linear regression models including each scale (WAI, WPAI, EuroQoL, BDI-II, PSQI), in turn, as dependent variable, and disease status as independent variable (people with MS vs. controls). Then, correlates of working ability (WAI), productivity and activity impairment (WPAI) were evaluated using linear regression models including each of these scales (WAI, WPAI), in turn, as dependent variable, and potential correlates (EuroQoL, BDI-II, PSQI), in turn, as independent variable; we also included an interaction term between disease status (people with MS vs. controls) and potential correlates (EuroQoL, BDI-II, PSQI), in turn, to evaluate changes in these associations between people with MS and controls.

Correlates of MS-related perceived difficulties in work-related tasks were evaluated using linear regression models including MSQJob as dependent variable, and each additional clinical variable (disease duration, EDSS, walking impairment on EDSS, clinical subtype, DMT, EuroQoL, FSS, SDMT, CVLT, BVMRT, cerebral functional system), in turn, as independent variable.

Univariate linear regression models were preliminarily run to evaluate associations between main variables of interests and study endpoints, in turn. We selected age, sex, education, and type of contract as covariates, that were included in all statistical models.

Distribution of variables and residuals was checked using both graphical and statistical methods. Results are reported as coefficients (Coeff), 95% confidence intervals (95% CI), and *p*-values, as appropriate. Statistical analyses were performed using Stata 17.0.

## Result

We included 71 people with MS and 71 controls. Demographics and occupational history in subjects with MS and controls are reported in Table 1. MS workers had similar age, when compared with controls (*p* = 0.61), but were more frequently females (*p* < 0.01), less educated (*p* = 0.01), more working disabled (*p* < 0.01), less exposed to occupational risk factors (*p* < 0.01), and more limited in fitness to work (*p* = 0.01) (Table 1).

**Table 1:** Demographics and occupational history in people with MS and controls.

		**People with MS (*n* = 71)**	**Controls (*n* = 71)**	***p*-value**
Age, mean ± SD		41.7 ± 9.4	42.6 ± 11.9	0.61
Sex, females (%)		42 (59.1%)	24 (33.8%)	<0.01
Education	Degree	36 (50.7%)	53 (74.6%)	0.01
	High school	33 (46.5%)	16 (22.6%)	
	Primary school	2 (2.8%)	2 (2.8%)	
Working disability status, *n*		68 (95.7%)	1 (1.4%)	<0.01
Working disability, percent		43.7 ± 35.1%	1.0 ± 8.4%	<0.01
Contract	Permanent	48 (67.6%)	40 (56.3%)	0.08
	Temporary	11 (15.5%)	22 (31.0%)	
	Self employed	12 (16.9%)	9 (12.7%)	
Work classification*	Law makers, businessmen, and managers	13 (18.3%)	10 (14.1%)	0.69
	Intellectual, scientific and high-specialization work	9 (12.7%)	7 (9.9%)	
	Technical work	9 (12.7%)	5 (7.0%)	
	Office executive work	3 (4.2%)	3 (4.2%)	
	Qualified work in commercial activities and services	18 (25.3%)	19 (26.8%)	
	Craftsmen, skilled workers, and farmers	15 (22.6%)	24 (33.8%)	
	System operators, workers of fixed and mobile machinery, and vehicle drivers	0 (0%)	0 (0%)	
	Unqualified work	3 (4.2%)	3 (4.2%)	
	Armed forces	0 (0%)	0 (0%)	
Occupational risks	Physical	15 (21.2%)	9 (12.7%)	<0.01
	Ergonomic	17 (23.9%)	23 (32.4%)	
	Biological/Chemical	3 (4.2%)	30 (42.2%)	
	None	36 (50.7%)	9 (12.7%)	
Limitations in fitness for work		11 (15.5%)	2 (2.8%)	0.01

*P*-values are shown from t-test, chi-square test and Fisher's exact test, as appropriate. MS, multiple sclerosis; SD, standard deviation.

^*^Work classification refers to the Italian Institute of Statistics (ISTAT – Nomenclatura e classificazione delle Unità Professionali).

Working impairment (e.g., ability, productivity and activity) of people with MS and controls, and their associations with clinical features (e.g., quality of life, depression, quality of sleep) are reported in Table 2. People with MS had worse WAI (Coeff = −5.47; 95% CI = −7.41, −3.53; *p* < 0.01), EuroQoL (Coeff = −4.24; 95% CI = −17.85, −6.50; *p* < 0.01), BDI-II (Coeff = 3.99; 95% CI = 2.37, 7.01; *p* < 0.01), and PSQI (Coeff = 4.74; 95% CI = 3.13, 7.61; *p* < 0.01), compared with controls, while no difference in WPAI was found (Coeff = −1.62; 95% CI = −7.82, 4.57; *p* = 0.60). EuroQoL, BDI-II, and PSQI were associated with both WAI and WPAI (all *p* < 0.01); however, when evaluating differences between people with MS and controls in these associations using interaction terms, only the association between EuroQoL and WAI was less strong in people with MS, when compared with controls (*p* < 0.01) (Table 2).

**Table 2:** Working impairment in people with MS and controls, and their associations with clinical features.

	**People with MS (*n* = 71)**	**Controls (*n* = 71)**	**Coeff**.	**95% CI**		***p*-value**
				**Lower**	**Upper**	
WAI	37.5 ± 6.4	43.7 ± 4.7	−5.47	−7.41	−3.53	<0.01
WPAI	4.6 ± 12.6	3.9 ± 14.0	−1.62	−7.82	4.57	0.60
EuroQoL, percent	73.5 ± 16.9	85.5 ± 14.2	−4.24	−17.85	−6.50	<0.01
Association with WAI			0.15	0.10	0.21	<0.01
Interaction term WAI			0.15	0.04	0.25	<0.01
Association with WPAI			−0.32	−0.49	−0.14	<0.01
Interaction term WPAI			−0.99	−0.53	0.18	0.32
BDI-II	8.3 ± 7.5	3.1 ± 5.0	3.99	2.37	7.01	<0.01
Association with WAI			−0.47	−0.60	−0.35	<0.01
Interaction term WAI			−0.18	−0.28	0.23	0.85
Association with WPAI			0.75	0.30	1.20	<0.01
Interaction term WPAI			−0.80	−1.31	0.55	0.42
PSQI	9.5 ± 6.5	4.7 ± 5.7	4.74	3.13	7.61	<0.01
Association with WAI			−0.48	−0.61	−0.35	<0.01
Interaction term WAI			−0.10	0.41	−0.35	0.14
Association with WPAI			1.09	0.65	1.54	<0.01
Interaction term WPAI			−0.05	−0.92	0.87	0.95

Coefficients (Coeff), 95% confidence intervals (95% CI), and p-values are shown from linear regression models adjusted by age, sex, education, and type of contract. First, we evaluated differences between people with MS and controls; then associations between WAI/WPAI and EuroQoL/BDI-II/PSQI; and, finally, changes in these associations between people with MS and controls on an interaction term. BDI-II, Beck Depression Inventory II; EuroQoL, European Questionnaire for Quality of Life; MS, multiple sclerosis; PSQI, Pittsburgh Sleep Quality Index; WAI, Work Ability Index questionnaire; WPAI, Work Productivity and Activity Impairment Questionnaire.

Clinical features of workers with MS, and their associations with perceived work-related difficulties (MSQJob) are reported in Table 3. Worse MSQJob was associated with higher EDSS (Coeff = 5.22; 95% CI = 2.24, 7.95; *p* < 0.01), walking impairment (EDSS > 4.0) (Coeff = 5.59; 95% CI = 7.20, 23.97; *p* < 0.01), progressive disease subtype (Coeff = 14.62; 95% CI = 5.56, 23.69; *p* < 0.01), EuroQoL (Coeff = 4.63; 95% CI = 2.92, 6.35; *p* < 0.01), FSS (Coeff = 0.55; 95% CI = 0.38, 0.72; *p* < 0.01), CVLT (Coeff = −0.31; 95% CI = −0.52, −0.09; *p* < 0.01), and MS-related cerebral functional system involvement (Coeff = 4.42; 95% CI = 0.67, 8.22; *p* = 0.02) (Table 3).

**Table 3:** Clinical features of people with MS and associations with MSQJob.

		**People with MS (*n* = 71)**	**Coeff**.	**95% CI**		***p*-value**
				**Lower**	**Upper**	
MSQJob, percent		17.0 ± 12.6	n/a	n/a	n/a	n/a
Disease duration, years		10.5 ± 7.4	0.01	−0.59	0.68	0.98
EDSS, median (range)		2.0 (1.0–6.0)	5.22	2.24	7.95	<0.01
<3.5		59 (83.1%)	15.59	7.20	23.97	<0.01
>4.0		12 (16.9%)				
Clinical subtype	RRMS	61 (85.9%)	Ref.			
	Progressive (SPMS and PPMS)	10 (14.1%)	14.62	5.56	23.69	<0.01
DMT						
1st line	Dimethyl fumarate	10 (14.2%)	Ref.			
	Glatiramer acetate/Interferon	6 (8.4%)				
	Teriflunomide	1 (1.4%)				
2nd line	Alemtuzumab	8 (11.3%)	3.55	−3.71	10.83	0.33
	Fingolimod	11 (15.5%)				
	Natalizumab	28 (39.4%)				
	Ocrelizumab	7 (9.8%)				
EuroQoL, score		2.0 ± 1.6	4.63	2.92	6.35	<0.01
FSS		29.6 ± 14.5	0.55	0.38	0.72	<0.01
SDMT, adjusted score		50.0 ± 12.5	−0.15	−0.42	0.11	0.25
CVLT, adjusted score		48.6 ± 14.5	−0.31	−0.52	−0.09	<0.01
BVMRT, adjusted score		52.1 ± 13.5	−0.09	−0.33	0.15	0.45
Cerebral FS	0	57 (80.3%)				
	≥1	14 (19.7%)	4.42	0.67	8.22	0.02

Coefficients (Coeff), 95% confidence intervals (95% CI), and p-values are shown from linear regression models adjusted by age, sex, education, and type of contract. BVMRT, Brief Visuospatial Memory Test-Revised; CVLT, California Verbal Learning Test; DMT, disease modifying treatment; EDSS, expanded disability status scale; EuroQoL, European Questionnaire for Quality of Life; FS, functional system; MS, multiple sclerosis; MSQJob, multiple sclerosis questionnaire for job difficulties; PPMS, primary progressive MS; RRMS, relapsing remitting MS; SDMT, symbol digit modalities test; SPMS, secondary progressive multiple sclerosis.

## Discussion

In the present study, we showed that, working ability, productivity and activity impairment are associated with quality of life, depressive symptoms, and sleep quality in both MS and age-matched controls, though overall working ability is lower in people with MS. In particular, determinants of perceived work-related difficulties in MS are disability, walking impairment, progressive symptoms, lower quality of life, fatigue and cognitive dysfunction. Of note, we specifically focused on employed people with MS, rather than on unemployment, and, thus, our work aims at increasing awareness on determinants of early working impairments, and at considering medications, rehabilitation, and specific working adaptations to tackle employability in clinical practice (16). The baseline level of work productivity is associated with work productivity trajectories over time (11), and, thus, variables associated with working difficulties should be identified and targeted as soon as possible during the course of the disease.

We showed that employed people with MS have lower working ability, when compared with controls, but are able to keep up with requested working productivity and activity, possibly thanks to the acknowledgment of working disability and subsequent arrangements (e.g., reduced or modified exposure to selected occupational risk factors). In previous studies, there was poor awareness on the tools to assist people with MS in retaining employment (27), while this does not seem the case in our population. Over the disease course, there is a progressive reduction of occupational activities, with up to 50% patients with MS being unemployed within 10 years from disease onset (8, 9, 27–29). As such, the employability of workers with MS should be preserved by adaptation and prevention of working conditions (e.g., ergonomic and technical aspects of the workplace) based on the individual clinical manifestations of the disease (11). In a recent survey, Italian occupational physicians reported on difficulties in rating fitness to work in people with MS (30), and, thus, we hope that our study will raise awareness on the opportunity to design and implement special and reasonable accommodations for MS workers in order to meet their individual needs, according to their clinical features.

Among the novelties of our study, we included both people with MS and controls, and showed that quality of life, depressive symptoms and quality of sleep were worse in people with MS, when compared with controls, but were equally associated with working activity and capacity (productivity and ability). These results suggest that, though quantitatively different, people with MS and controls share qualitatively similar correlates of working impairment. As such, early identification and clinical management of worsening quality of life, depression and sleep, along with subsequent working adaptations, may contribute to improve productivity and to retain employment in individuals with MS, as well as in otherwise healthy workers (10, 11).

Looking at MS-specific factors, in our study, self-perceived difficulties in work-related tasks were associated with higher disability (especially walking impairment), progressive disease subtype, fatigue and cognitive dysfunction, especially in relation to verbal learning and memory. These results are in line with previous studies showing associations between unemployment and mood changes (11, 13), fatigue (8, 13, 28), motor disability (8, 13, 28, 31, 32), and cognitive dysfunction (32). Intriguingly, we specifically focused on workers with MS, and decided to evaluate determinants of perceived working difficulties, which were ultimately not different from actual correlates of unemployment. In keep with this, previous studies showed associations between work difficulties and mood changes (14, 33, 34), fatigue (11, 14, 34), motor disability (11, 14, 33), and cognitive dysfunction (11, 14, 35). In a previous study also using the BICAMS, attention and processing speed (SDMT) was associated with working ability (35), while we found an association for verbal learning and memory (CVLT). While we acknowledge that the SDMT is a marker of overall cognitive function in MS (36), in our study, we computed the cerebral FS score based on overall BICAMS results, which also reflects cognitive impairment (26), and was associated with working difficulties. Not least, working difficulties can be associated with a variety of cognitive deficits based on the actual tasks. We cannot exclude a similar association in controls as well, but unfortunately did not have availability of cognitive variables.

Our study is limited by the single-center design and the relatively small sample size, though sufficiently powered to show consistently significant associations. Controls were recruited based on the age range, which is a main determinant of employability; however, cases and controls were not balanced in sex and education, which were included as covariates in the statistical models. As such, notwithstanding statistical adjustments, we have to acknowledge the risk of bias coming from sex and education differences. Also, our cross-sectional design does not allow causal inference, nor the evaluation of longitudinal changes in working difficulties, also in relation to treatments (20). Generalizability of our results is definitely limited to countries with similar working retention policies and universal healthcare coverage (e.g., Europe, Canada) (17, 37).

In conclusion, our study encourages the early identification and management of clinical features potentially causing an impairment of working ability in people with MS, along with the implementation of individually targeted prevention and protection measures on the workplace. Based on our results, MS specialists should primarily consider disability, fatigue, depression, and cognitive impairment, and liaise with occupational physicians to identify the most suitable arrangements, counseling programs and strategies to support workers with MS. In the future, multidisciplinary patient/worker management teams, including MS specialists, occupational physicians and other healthcare professionals (38, 39), should be considered to protect the health and the employment of vulnerable people with MS.

## Data availability statement

The raw data supporting the conclusions of this article will be made available by the authors, without undue reservation.

## Ethics statement

The studies involving human participants were reviewed and approved by Federico II University Ethics Committee. The patients/participants provided their written informed consent to participate in this study.

## Author contributions

MM and LF contributed to data collection, data analysis and interpretation, manuscript preparation, and revision. VB and II contributed to data interpretation, manuscript revision, and coordination. RP and MT contributed to data analysis and interpretation and manuscript revision. FFa, FFi, MF, RL, and LR contributed to data collection, database preparation, and manuscript revision.
